# Glycosaminoglycans from Co-Products of «*Scyliorhinus canicula*»: Extraction and Purification in Reference to the European Pharmacopoeia Requirement

**DOI:** 10.1186/s12575-019-0113-1

**Published:** 2020-01-02

**Authors:** Nawras Talmoudi, Noureddine Ghariani, Saloua Sadok

**Affiliations:** 1 Blue Biotechnology & Aquatic Bioproducts Laboratory (B3Aqua)-Institut National des Sciences et technologies de la Mer (INSTM), 28, street March 2, 1934 –Salammbô, 2035 Tunis, Tunisia; 2 Faculty of Mathematical, Physical and Natural Sciences of Tunis-University of El Manar, Tunis, Tunisia; 3 TERIAK pharmaceutical companies, Industrial Zone Cheylus, 1111 JEBEL OUEST, Tunisia

**Keywords:** *Scyliorhinus canicula*, Glycosaminoglycans, Chondroitin sulfate, Dermatan sulfate, Electrophoresis, FT-IR, HPLC, RMN

## Abstract

**Background:**

Glycosaminoglycans (GAGs), including hyaluronic acid (HA), dermatan sulfate (DS) and chondroitin sulfate (CS) are essential components of the bone and cartilage tissues. CS isolated from the cartilage tissue of various animals has found application in pharmaceuticals, cosmetics and food industries. In the first part of the present work, three methods were used and compared to extract and purify glycosaminoglycans (GAGs) from the cartilage powder of a local cartilaginous marine species «*Scyliorhinus canicula*». One of these GAGs, chondroitin sulfate (CS), will be exploited for the development of an anti-osteoarthritis generic at the request of a collaborative pharmaceutical industry. Thus this active ingredient must meet the requirements and tests described by the European Pharmacopoeia (Ph. Eur.). These tests are treated in the second part of this work.

**Results:**

Among the three methods that have been applied in the present work, in order to optimize the best process for GAGs preparation, enzymatic hydrolysis with papain followed by deproteinisation using trichloroacetic acid (TCA) was found the best one. The separation of the extracted GAGs using agarose gel electrophoresis, and the identification of bands by Fourier Transform Infrared (FT-IR) Spectroscopy, revealed that the cartilage GAGs of *« Scyliorhinus canicula»* are exclusively chondroitin sulfate (CS) and dermatane sulfate (DS), with proportions of 12.889 and 87.111% respectively, and that CS is of type C. The extraction technique with papain provides a product with GAGs content of around 90%. The TCA deproteinisation yielded the lowest level of protein (2.8%) in the extracted GAGs, less than 3%, which is the standard required by the European Pharmacopoeia (Ph. Eur.).

Cetylpyridinium chloride (CPC) assay suggests that the titration technique, although is introduced by the Ph. Eur. for the determination of CS content, is not an accurate method, and that the values obtained by the optimized and validated HPLC method, described in this work, are more exact.

**Conclusion:**

The extracted and purified active ingredient is perfectly conform to the tests described by the Ph. Eur. The results suggest that the co-product of *Scyliorhinus canicula* would be a perfect source of molecules of pharmacological interest, obtained by a simple and non-agressive process.

## Background 

Cartilage is a pre-eminent by-product of great value in the nutraceutical and pharmacological fields. Glycosaminoglycans extracted from the cartilage have proved to be among the most important compound [1]. GAGs are long linear polysaccharides, structurally very complex, polar and negatively charged molecules [2]. GAGs have created a new research potential because of their diversity and their very large structural and functional varieties. They include hyaluronic acid (HA), Dermatan sulfate (DS) and chondroitin sulfate (CS) as well as heparin (Hep), heparan sulfate (HS) and kertan sulfate (KS). Clinically, sulfated GAGs are used as chondro-protective drugs in all types of arthritis in humans [3]. CS represents an anti-inflammatory activity [4, 5]. It has been reported that oral administration of CS decreases osteoarthritic symptoms [6, 7]. The use of anti-osteoarthritis drugs becomes the concern of the pharmaceutical companies. Indeed, clinical applications require highly purified CS compared to cosmetics or dietary supplements. Analytical protocols for determining the structure, physicochemical characteristics, and purity of each type of CS found in pharmaceuticals have been established in different ways.

A commonly used enzyme, papain, has been tested to release GAGs [8]. Isolation of chondroitin sulfate from cartilage of the dogfish in sodium acetate buffer solution with papain was also performed. Digestion is often stopped by denaturing the enzyme at 100 °C for 15 min [9]. Enzymatic treatment with two combined enzymes (alkalase and flavourzyme) has been also tested and showed a better GAG yield as well as a significant reduction in treatment time [10]. The unmilled shark cartilage was extracted with boiling water and digested with pancreatic enzyme [11, 12]. GAGs were also released from cartilage by activation of endogenous enzymes (autolysis) [13, 14] and approximately 70% of total CS was obtained. For efficient GAGs extraction, tissue degreasing with organic solvents and deproteinization with trichloroacetic acid is suggested as an important process [15–17].

The subsequent phase of alcoholic treatment is a crucial step for the selective precipitation of CS from the hydrolyzate [9, 12]. Fractionated mixtures of GAGs by sequential precipitation with methanol, ethanol or propanol give the same behaviour. Therefore, sequential precipitation with ethanol seems to be the best method for fractionating GAGs mixtures because this alcohol is considered as a solvent for substances intended to be in contact or consumed by human [18]. GAGs were fractionated using isopropyl alcohol containing 2% NaCl [10, 14]. Chromatographic methods were also used but they are time-consuming and costly process for industry [15, 19].

The resulting GAG fraction is further purified by membrane filtration to remove salt and low molecular weight materials. Other studies reveal the high efficiency of the 30 kDa UF-DF system as the final step in CS retention and recovery and protein removal from *S. canicula* co-products [20]. Purification also involved dialysis against deionized water to provide the crude polysaccharide fraction that will be lyophilized [21]. Analytical methods for the determination of the CS content and the presence of impurities are necessary for the quality control of the nutraceutical and pharmaceutical raw materials as well as the finished marketed products. The analytical methods used involve the identification by high performance liquid chromatography [22], the detection of impurities by agarose gel electrophoresis [23, 24] and photometric titration with cetylpyridinium chloride [25]. However, these methods are not specific and are applied to total GAGs. More specific methods including Fourier Transform Infrared spectroscopy [10, 26] and nuclear magnetic resonance (NMR) [27] were used.

Our project involves the optimisation of an extracting and purifying GAGs method from the cartilage of a local cartilaginous fish « *Scyliorhinus canicula* ». One of the molecules, the CS, is required for the development of an anti-osteoarthritis treatment, and must meet the requirements and tests described by the European Pharmacopoeia [28] and listed in the CS monograph [29]. Analytical protocols for determining physicochemical characteristics and purity of the CS molecule are established. Several methods are commonly used for the extraction and segregation of GAGs [15, 30–32]. In this paper, all the methods of the monograph have been applied in addition to other methods that we suggested more accurate. Isolated CS was identified by FT-IR and analysed by HPLC. Agarose gel electrophoresis and NMR were performed. Statistical analysis of the different results was carried out using IBM SPSS Statistics software.

## Results

### Physico-Chemical Characteristics of Fractions I, II and III of Total GAGs

Considering the resulting fractions of total GAGs obtained from the powered material of the starting matrix (20 g of cartilage powder), fractions I, II and III are obtained as explained. The results of qualitative and quantitative physicochemical tests applied are summarized in Table 1.

**Table 1:** Physicochemical characteristics of fractions I, II and III of total GAGs

TESTS	TCA	ENZ.DIG	ALKA.HYDRO	ASPECT	SOLUBILITY	WEIGHT (g)	YIELD (%)	pH	L.O.D (%)	SODIUM.R	CHLORIDE.TEST
Fractions											
FRACTION I	+	+	–	Wh.Hyg.Pow	Wat.Solu Act/Eth.Ins	1.35	6.75	6.65	10.8	COMPLIANT	COMPLIANT
FRACTION II	–	+	–	Wh.Hyg.Pow	Wat.Solu Act/Eth.Ins	3.25	16.25	6.68	–	COMPLIANT	COMPLIANT
FRACTION III	–	+	+	Wh.Hyg.Pow	Wat.Solu Act/Eth.Ins	3.35	16.75	6.81	–	COMPLIANT	COMPLIANT

(+): added

(−): not added

*ENZ.DIG* Enzymatic digestion

*ALKA.HYDRO* Alkaline hydrolysis

Trichloroacetic acid deproteinisation

*Wh.Hyg.Pow* white hygroscopic powder

*Wat.Solu* easily soluble in water

*Act/Eth.Ins* Insoluble in acetone and 96% ethanol

*L.O.D* loss on drying

*SODIUM.R* sodium reaction

Fractions I, II and III are milled after purification and lyophylization. They have an appearance of hygroscopic white powder, very soluble in water and insoluble in organic solvents (alcohol, ethanol...). The pH is 6.65, 6.68 and 6.81 for fractions I, II and III respectively. The sodium reaction and the chloride test are also conform to standards. The loss on drying (LOD) applied to fraction I is compliant too and is about 10.8% (the reason for choosing this fraction for this test and for other tests is mentioned below).

Fraction I has the lowest weight (1.35 g) and therefore the lowest yield (6.75%). This fraction was protein-free by adding TCA, and seems to be the purest one. The alkaline hydrolysis associated to the enzymatic digestion used for preparation III slightly increased the yield of GAGs fraction (16.75%) compared to that of fraction II where only enzymatic digestion was used (16.25% of total GAGs). Alkaline hydrolysis appears to be useless, as it has no effect on the performance of GAGs extraction and considering the additional chemical step used.

The other physicochemical tests are treated separately in detail below.

### Protein Determination

In the adopted method, the protein-Cu2^+^ of the Folin reagent complex develops a blue color, which is proportional to the level of the proteins in the GAGs solutions. The concentration (CC) of proteins in each fraction is given in Table 2.

**Table 2:** Protein concentration (CC) of fractions I, II and III of total GAGs

Descriptive statistics									
	N	CC (mg/ml)	Minimum	Maximum	Sum	Average (%)		Standard deviation	Variance
	Statistics		Statistics	Statistics	Statistics	Statistics	Std. Error	Statistics	Statistics
FI	4	0.026	2.76	2.98	11.50	2.8750	.04664	.09327	.009
FII	4	0.053	5.58	6.05	23.41	5.8525	.11807	.23613	.056
FIII	4	0.058	6.27	6.65	25.72	6.4300	.09557	.19114	.037
N valide (listwise)	4								

The obtained results show that TCA treatment significantly reduces the level of contaminating proteins. Only fraction I treated with TCA showed a concentration lower than 3% (2.8% of contaminating proteins for fraction I against 5.8% for fraction II and 6.4% for fraction III). This value is in accordance with that required by the European Pharmacopoeia. These results reinforced those of Table 1 where the yield of Fraction I was the lowest compared to fractions II and III, which have not been deproteinized.

### Intrinsic Viscosity

The viscosity test is carried out only on fraction I of total GAGs. The choice of this fraction for many tests was based on the fact that, unlike fractions II and III, fraction I is conform to the standard protein value of the European Pharmacopoeia [28]. According to this test, intrinsic viscosity must range between 0.01 and 0.15 m^3^/kg. The results are summarized in Table 3. Intrinsic viscosity of fraction I is 0.12 m^3^/kg and was in accordance with the standards of the Ph. Eur. This is further evidence that fraction I, extracted with papaine digestion and deproteinized with TCA, meets the requirements.

**Table 3:** Viscosity values of fraction I of total GAGs

Flow time (s)	Relative viscosity	Specific viscosity [ηs]	C (kg/m3)	Intrinsic viscosity [η]
124.393	1.32	0.32	26.146	0.120

### Study of Fractionated GAGs

Physico-chemical tests were applied to fractions I, II and III of total GAGs (represented in Table 1) and not to fractionated GAGs for ease of handling. Only the yield of the fractions sequentially precipitated with ethanol (fractions A to J) was performed. The results are represented in Table 4.

**Table 4:** Yield of GAGs fractions extracted by sequential precipitation with ethanol

FRACTIONS	A (0.2 V)	B (0.4 V)	C (0.6 V)	D (0.8 V)	E (1 V)	F (1.2 V)	G (1.4 V)	H (1.6 V)	I (1.8 V)	J (2 V)
WEIGHT (g)	0.04	0.055	1.5	0.13	0.055	0.045	0.006	traces	traces	traces
YIELD (%)	0.2	0.275	7.5	0.65	0.275	0.225	0.03	–	–	–

Total GAGs fraction was obtained following the same extraction protocol applied to fraction I described previously, and which turned out to be the purest form after deproteinization with TCA. For 20 g of raw material used, followed by fractionation of the total GAGs obtained with ethanol, the highest yield was observed in fraction C (7.5%) where a large amount of GAGs was extracted with 0.6 volumes of added ethanol. The amount of the extracted GAGs decreased sharply at 0.8 volumes of added ethanol (0.65%) then gradually dropped to reach 0.03% at 1.4 volumes of added ethanol. From 1.6 volumes of ethanol added, the precipitated fractions were in the form of traces. No GAGs extracted beyond two volumes of added ethanol, and the extraction suspension, which had initially a very viscous appearance with dark yellow colour, becoming very clear and light.

### Identification of GAGs and Impurities by Agarose Gel Electrophoresis: Electrophoretic Profiles of Fractions I, II and III of Total GAGs and Fractions a to J of Fractionated GAGs

Agarose gel electrophoresis was applied to fractions I, II and III of total GAGs as well as fractionated GAGs for identification. The technique made it possible to determine the degree of safety of the obtained fractions.

**Fig 1:**
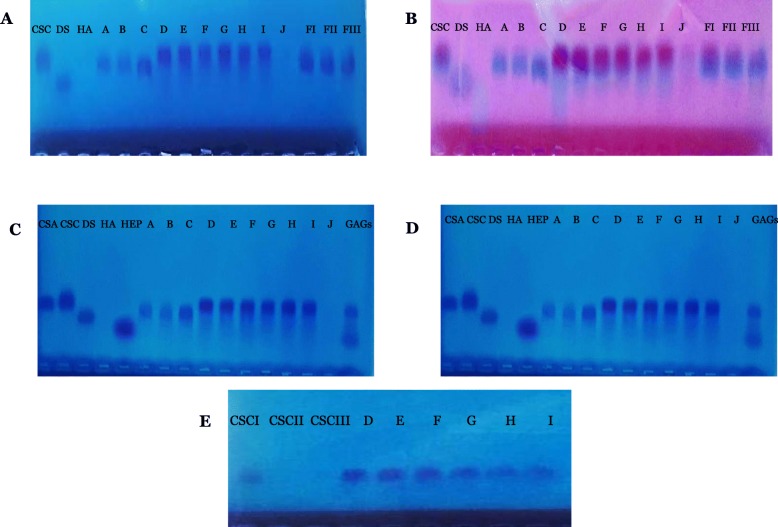
**a, c** Electrophoretic profile of fractionated GAGs staining with toluidine blue. **b, d** Electrophoretic profile of fractionated GAGs staining with toluidine blue/stains-All. (CSA) Chondroïtine sulfate A standard. (CSC) chondroitin sulfate C standard. (DS) Dermatan sulphate standard. (HA) Hyaluronic acid standard and (HEP) heparin standard. (A-J) extracted fractions with 0.2v to 2v of ethanol. (GAGs) total GAGs standards. (FI, FII, FIII) fractions I, II and III of total GAGs. **e** Electrophoretic profile of fraction E of pure CSC. Standard of CSCI (30 mg/ml), CSCII (0.6 mg/ml) and CSCIII (0.3 mg/ml). (D-I) Extracted fractions corresponding on pure CS fractions obtained with 0.8v-1.8v of ethanol

Fig. 1a and b represent the electrophoretic profiles of fractionated GAGs molecules as well as fractions I, II and III of total GAGs compared to standards of chondroitin sulfate C (CSC), dermatane sulfate (DS), and hyaluronic acid (HA). Fig. 1c and d represent fractionated GAGs compared with standards of chondroitine sulfate A (CSA), chondroitin sulfate C (CSC), dermatane sulfate (DS), hyaluronic acid (HA) and heparin (HEP). Staining with toluidine blue alone (Fig. 1a and c) and after combined staining with toluidine blue and stains-all (Fig. 1b and d), fractions I, II and III showed the presence of the two related molecules: CSC and DS, with a predominance of the latter. No other molecule was visible on the profile; the composition of the samples as well as the degree of purity seemed to be similar for the three extractions according to this technique. Indeed, despite the large amount of sample injected into the wells (10 μl), no impurity detected.

Electrophoretic profiles of the different GAGs fractions obtained after sequential ethanol precipitation and staining with toluidine blue alone (Fig. 1a and c) showed that fractions A, B and C (precipitated with 0.2volume (v); 0.4v and 0.6v of ethanol) migrate at the same level as the standard of DS. Three fractions therefore corresponded to DS. Fractions D to J (precipitated with 0.8v to 2v of ethanol) showed the same electrophoretic behaviour as that of standard of CSC. Even after stains-all stains (Fig. 1b and d), the colour of the latter intensified and took a purple hue, meanwhile CSA standard did not stain further after combined coloration with a lesser migration. These fractions therefore corresponded to the CSC. The additional stains-all staining which aims to reveal the presence of hyaluronic acid (molecule unable to bind toluidine blue and therefore cannot be revealed by the latter), shows that this molecule was absent in this extract. The fraction corresponding to 2% of ethanol added contains traces of CSC. Beyond 2% of ethanol added, no precipitation occurred. This suggests that in the cartilage of the «*Scyliorhinus canicula*» species, all GAGs molecules are completely extracted at two volumes of ethanol, and are exclusively CSC and DS. Indeed, no band of the fractions corresponds to the standards of HA and Hep.

### Qualitative Analysis of GAGs Extracts

#### Electrophoretic Profile of Pure CSC Molecule: Revelation of Related Substances According to the European Pharmacopoeia

Fig. 1e shows the pure CSC fractions obtained after sequential ethanol precipitation (Fraction D to I). The concentrations and the electrophoretic conditions are carried out according to the assay technique described in the European Pharmacopoeia. The latter suggests that, if secondary bands appear in the electropherogram obtained with the test solution, none of them should be more intense than the band of the electropherogram obtained with the CSC control solution of 0.3 mg/ml. Given the concentrations and dilutions performed, the sharpness of the bands suggests that the CSC molecules obtained after TCA deproteinization and ethanol fractionation did not contain impurities, and therefore meet the purity criteria required by the Ph. Eur [28].

In electrophoresis, and due to variations in the sulfation degree and molecular weight, the mobility of standards of GAGs was not necessarily the same as that of the corresponding GAGs samples prepared from a different source. Therefore, the identification of GAGs detected by electrophoresis required additional analysis. The following tests are then performed.

#### Identification of GAGs by Fourier Transform Infrared (FT-IR) Spectroscopy

The first IR spectra of GAGs were published more than 60 years ago. The method was sensitive enough to characterize, differentiate, and classify GAGs types and subtypes despite their close molecular structures, and gives a complete “molecular fingerprint” of the latters. This method is fast, non-destructive and does not require external markers. After combination of spectroscopy with a microscope, it becomes highly sensitive and requires only small amounts of sample. The FTIR spectrum of a sample to be analysed as well as the standard of the same sample should be similar. The position of the peaks in terms of wave number (cm-1) in the optimal conditions must be identical and correlated [33].

**Fig. 2:**
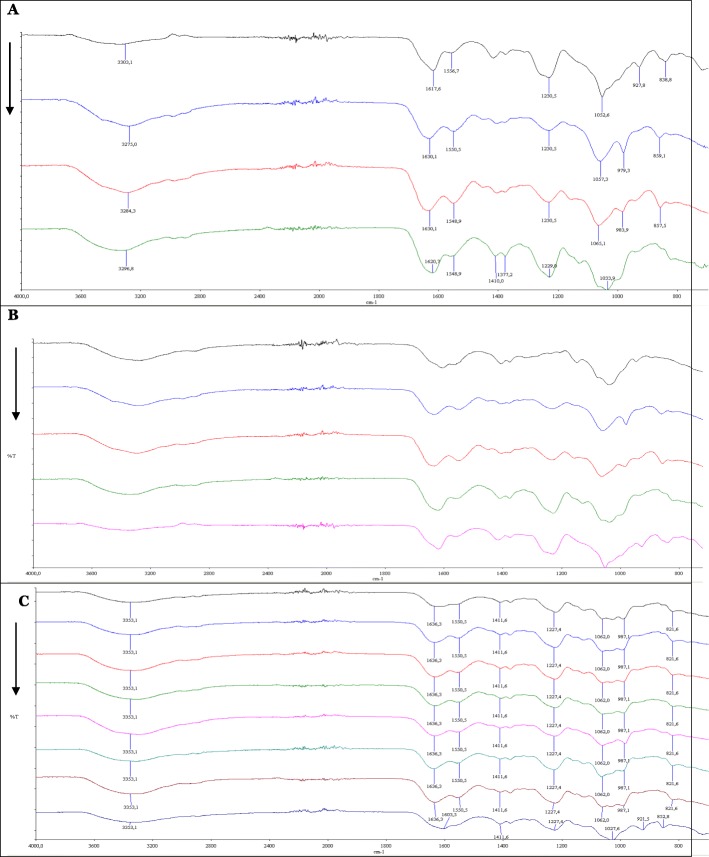
FT-IR profiles of standards and fractionated Glycosaminoglycans (GAGs). **a** FT-IR profiles of Dermatan sulfate (DS, USP grade) / fraction A (0.2 V) / fraction B (0.4 V) / fraction C (0.6 V). **b** FT-IR profiles of Hyaluronic acid (HA, USP grade) / fraction A / fraction B / fraction C / Dermatan sulfate (DS, USP grade). **c** FT-IR profiles of Chondroïtin sulfate C (CSC, USP grade) / Fractions D (0.8 V) to I (1.8v) / Chondroïtin sulfate A (CSA, USP grade)

The FT-IR profiles of different GAGs fractions obtained after ethanol precipitation as well as standards of CSA, CSC, DS and HA are superposed in order to identify these fractions. In the figure, GAGs profiles are listed from top to bottom. FT-IR profiles show that fractions A, B and C correspond to the DS after superposition of their profiles with those of DS (Fig. 2a) and HA (Fig. 2b). They also show that fractions D to I correspond to CSC after superposing their profiles with standards of CSA and CSC (Fig. 2c). The bands near 1610 and 1410 cm-1 were assigned, respectively, to the presence of the -COO-planar antisymmetric and symmetrical group vibrations at the C6 position of the disaccharide unit of uronic acid. The group around 1.250 cm-1 corresponded to the sulfate group (SO_3_^−^) present in all GAGs except hyaluronic acid, which is an unsulfated GAG. The region covering the range 1.100–1.000 cm-1 was associated with the vibration of the corresponding bonds at the C-O-C, C-C-C and C-C-O stretching of the GAG ​​molecules [34].

The results are compared with the electrophoretic analysis. These results are consistent with those obtained after identification by agarose gel electrophoresis. According to these results and from those shown in Table 4, we find that the cartilage of «*Scyliorhinus canicula*» is composed exclusively of two GAGs: CSC and DS, with a dominance of DS whose content reaches 87.111% whereas CSC covers 12.889%. From Table 4, we deduce that total GAGs fraction represents 9.155% of raw material and is composed of 7.975% of DS and only 1.18% of CSC (Table 5).

**Table 5:** Proportions of total GAGs, CS and DS fractions

% of total GAGs from cartilage powder (20 g)	% of DS from cartilage powder (20 g)	% of CS from cartilage powder (20 g)	% of DS from total GAGs fraction	% of CS from total GAGs fraction
9.155	7.975	1.18	87.111	12.889

#### Identification of GAGs by Nuclear Magnetic Resonance (NMR) Spectrum

The nuclear magnetic resonance (NMR) technique is a multidimensional spectroscopy, which has become in recent years the most important tool for the characterization of GAGs. This is mainly due to its high sensitivity and high accuracy, thus the possibility of in-line coupling with separation techniques such as liquid chromatography or capillary electrophoresis [27, 35].

**Fig. 3:**
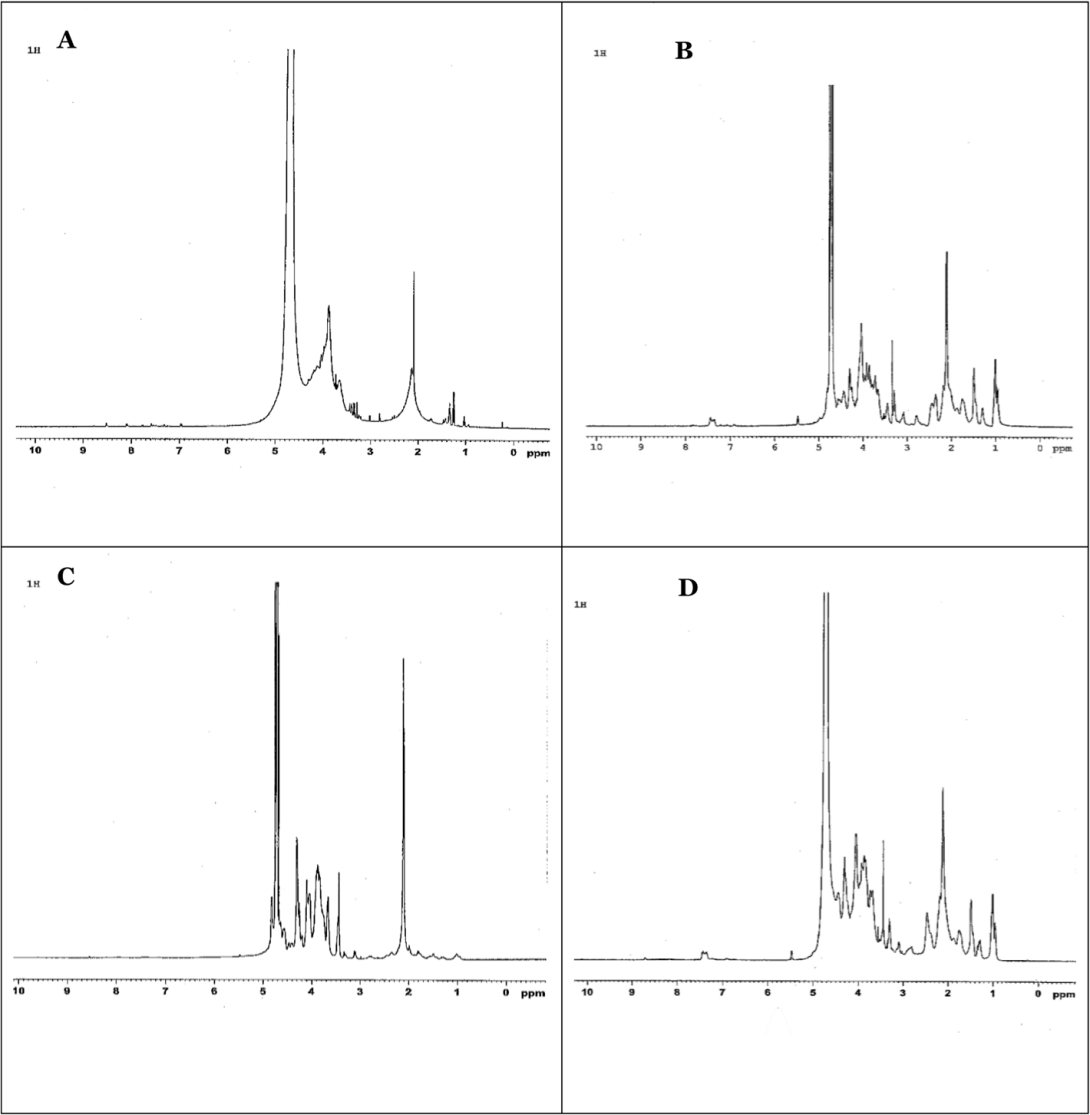
Nuclear magnetic resonance (NMR) profiles of standard and fractionated Glycosaminoglycans (GAGs). **a** NMR profile of Dermatan sulfate (DS, USP grade). **b** NMR profile of fraction C (0.6 V ethanol). **c** NMR profile of Chondroitin sulfate C (CSC, USP grade). **d** NMR profile of fraction D (0.8 V ethanol)

NMR profile of DS, fraction C, CSC and fraction D are represented in Fig. 3a, b, c and d simultaneously. NMR profile of DS standard was compared to fraction C obtained with 0.6v of ethanol added, which supposed to be DS, and NMR profile of CS standard was compared to fraction D obtained with 0.8v of ethanol added, which supposed to be CS. The obtained results show a high conformity of the compared profiles which reinforces both the electrophoresis and the FT-IR results. In the same time, NMR profile of CS revealed low number of sulphate group on hydroxyl sites of the GAG and proved that the obtained GAG was devoid of toxic motifs (OSCS) as discussed in another study [35].

### Quantitative Analysis of GAGs Samples

#### Photometric Determination of GAGs with Monohydrate Cetylpyridinium Chloride (CPC)

**Fig. 4:**
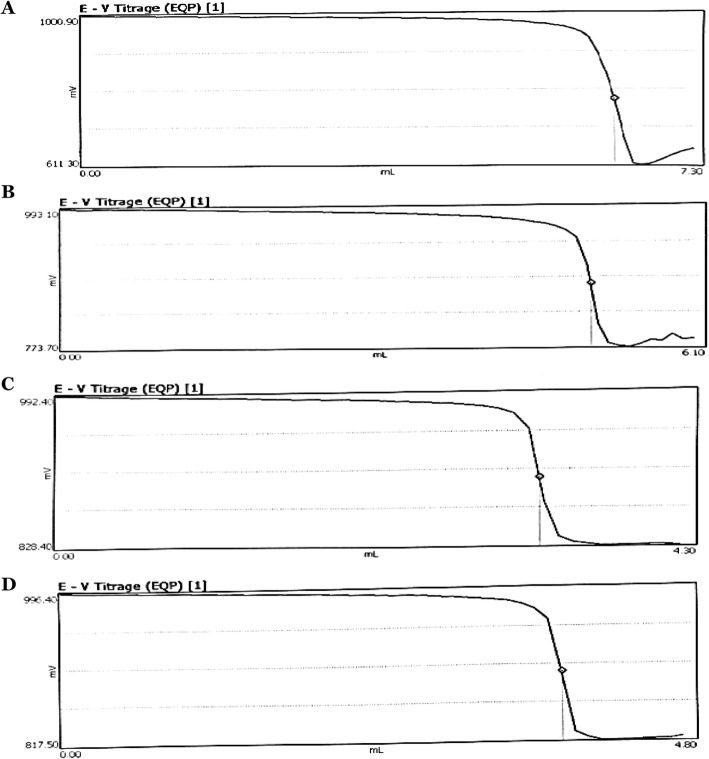
Cetylpyridinium chloride (CPC) titration of extracted chondroïtin sulphate and (USP) grade chondroïtin sulfate. **a** CS (USP grade) titration with CPC. **b** CS titration of fraction I with CPC. **c** CS titration of fraction II with CPC. **d** CS titration of fraction III with CPC

Titrimetric determination of CS with CPC is shown in Fig. 4a, b, c and d. The amount of CS estimated from fractions I, II and III (Fig. 4b, c and d successively) of total GAGs was compared to that of the USP grade CS (Fig. 4a). The percentage value of CS content was calculated according to the formula cited above. The CS content is 96.55% for the TCA-purified sample I (Fig. 4b), 53.05% for the sample II with no TCA added for deproteinization (Fig. 4c), and 60.00% for sample III that was obtained after enzymatic and alkaline hydrolysis, and without addition of TCA (Fig. 4d). TCA significantly increased the degree of purity of the extract and alkaline hydrolysis associated with enzymatic hydrolysis slightly increased the CS content. For all three samples, the extraction method applied to the first fraction seemed to be the most appropriate for obtaining pure CS sample.

According to other studies, CPC titration can give positive results for all anions of large molecules, such as proteins and surfactants. In this test, and considering that fractions I, II and II contain both CS and DS, it is clear that the CPC assay does not differentiate them and the values ​​obtained are those of the combined CS-DS chains. For this purpose, it was question of proceeding to another assay, which would be more specific and more reliable. All these informations led us to apply a simple method to identify and then to quantify intact and separated GAGs.

#### Identification and Quantification of GAGs by High Performance Liquid Chromatography (HPLC)

**Fig. 5:**
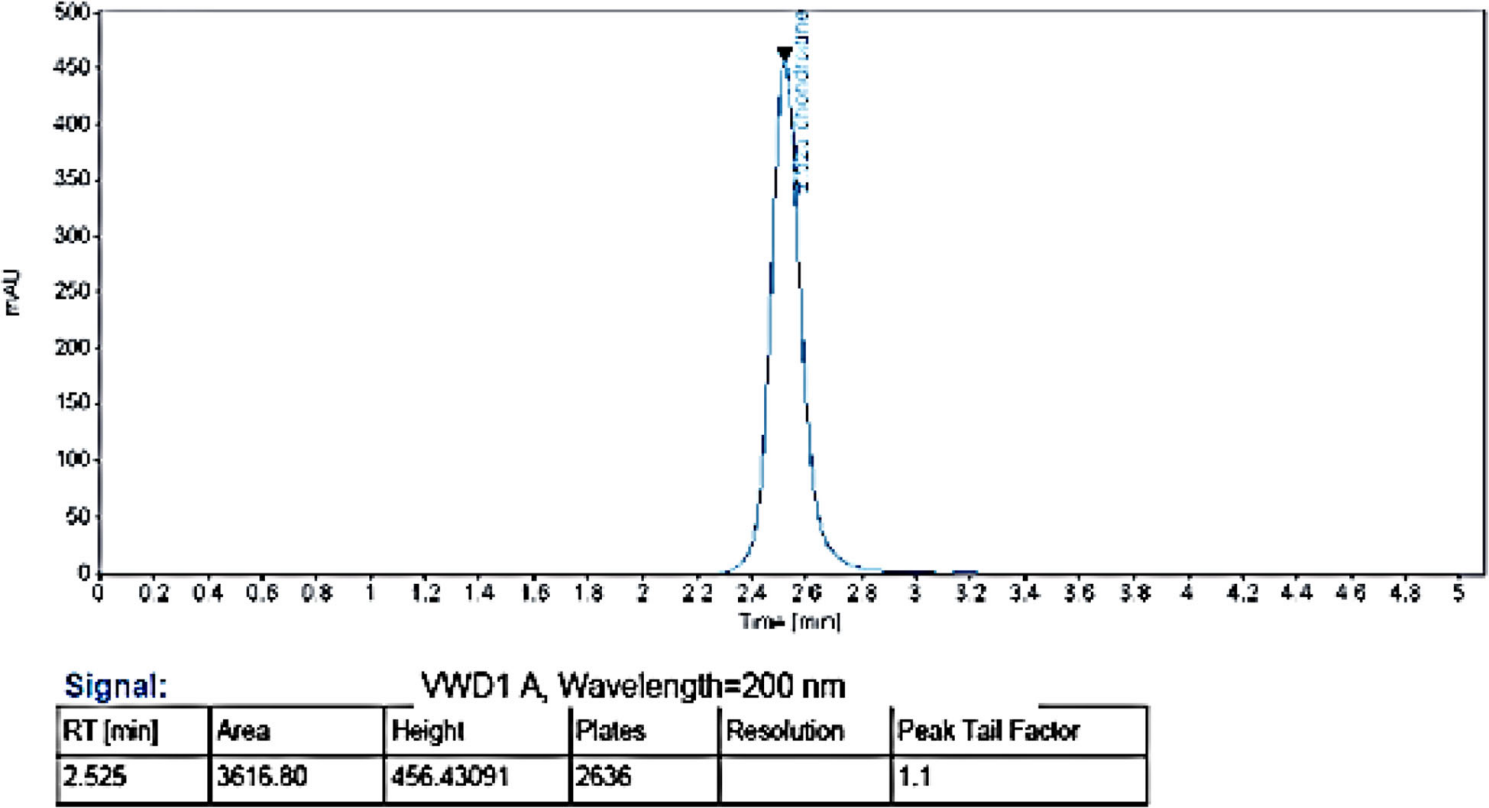
Typical chromatogram of standards of CS and DS according to the HPLC method

Analytical methods designed to quantify CS must be selective in the presence of other GAGs, whose chemical structures are very similar or even related. The present method was optimized then validated in our laboratory, and was adopted as a routine test for the determination of CS content in each obtained extracts. It is a direct method that does not require prior depolymerisation of GAGs with digestion enzymes (chondroïtinases) which are very expensive. Although it has been applied for the determination of CS content in solutions in other studies [22], we conclude that the method proved unspecific. Standards of CS and DS are elected at the same retention time (2.53 ± 0.01). The method cannot differentiate isomers, but it can still be used if prior identification of the polysaccharides to be quantified is performed. Electrophoretic profiles as well as FT-IR profiles allowed the identification of the different fractions of isolated GAGs after sequential precipitation with ethanol. These latter were quantified by the HPLC method. Fractions obtained at 0.2v, 0.4v and 0.6v of ethanol added (fractions A, B and C simultaneously) which represent DS are therefore quantified by standard of DS. Fractions obtained at 0.8v, 1v, 1.2v, 1.4v, 1.6v, 1.8v and 2v of ethanol added (fractions D to J) and which represent CSC are quantified by standard of CSC. A typical chromatogram of the two standards of GAGs (DS and CSC) is shown in Fig. 5. Contents of separated DS and CSC fractions are given in Table 6.

**Table 6:** HPLC Descriptive statistics of quantification of CS and DS fractions

FRACTIONS	N	Minimum content (%)	Maximum content (%)	Sum	Mean (%)	Standard deviation	Variance
Fraction A-DS (0.2 V)	2	43.70	46.80	90.50	45.2500	2.19203	4.805
Fraction B-DS (0.4 V)	2	59.50	60.50	120.00	60.0000	.70711	.500
Fraction C-DS (0.6 V)	2	90.60	90.60	181.20	90.6000	.00000	.000
Fraction D-CSC (0.8 V)	2	83.60	84.00	167.60	83.8000	.28284	.080
Fraction E-CSC (1 V)	2	81.80	82.10	163.90	81.9500	.21213	.045
Fraction F-CSC (1.2 V)	2	64.50	64.80	129.30	64.6500	.21213	.045
Fraction G-CSC (1.4 V)	2	57.50	57.60	115.10	57.5500	.07071	.005
Fraction H-CSC (1.6 V)	2	60.20	60.20	120.40	60.2000	.00000	.000
Fraction I-CSC (1.8 V)	2	58.90	59.00	117.90	58.9500	.07071	.005
Fraction J-CSC (2 V)	2	20.30	23.90	44.20	22.1000	2.54558	6.480
N valide (listwise)	2						

Fraction C obtained with 0.6 v of ethanol is the most concentrated in DS; the purity of this fraction is remarkable and the maximum amount of DS is extracted at this concentration of solvent (90.6%). Fraction D obtained at 0.8v of ethanol is the most concentrated in CSC (83.8%). CSC content decreases remarkably to 22.1% at fraction J when 2v of ethanol are added. These results reinforce those mentioned in Table 4. The yield of the two extracted GAGs, CSC and DS, are in complete accordance with their contents.

The GAGs content was also carried out for fractions I, II and III which represent combined CSC-DS chains and which have already been assayed for CPC. Values of both assays (HPLC and CPC) are represented in Table 7.

**Table 7:** Group statistic: Determination of CS - DS chains contents by HPLC and CPC methods

	Dosage type		N	Average	Standard deviation	Average standard error
Fraction I		Content(%) CPC Method	2	96.5500	8.41457	5.95000
		Content(%) HPLC Method	4	89.3000	.78740	.39370
Fraction II		Content(%) CPC Method	2	53.0500	.07071	.05000
		Content(%) HPLC Method	3	62.2667	1.41892	.81921
Fraction III		Content(%) CPC Method	2	60.0000	2.82843	2.00000
		Content(%) HPLC Method	4	65.4000	.89815	.44907

The obtained values are very close together, but the HPLC method always gives slightly lower grades. This confirms that the CPC assay was not specific. Fraction I was the most concentrated in CS-DS chains with highest purity (96.55% for CPC method and 89.30% for HPLC method). The purity decreases remarkably for fraction II when TCA is not added for deproteinization(53.05% for CPC method and 62.26% for HPLC method) and CS-DS content slightly increases in fraction III after combined enzymatic and alkaline hydrolysis (60.00% for CPC method and 65.40% for HPLC method). These results are consistent with those represented in Table 1, and which suggest that the yield of CS-DS chains slightly increases for fraction III after combined hydrolysis, and that fraction I deproteinised with TCA is the purest one. The T-test which was used to compare statistically the two methods are presented in Table 8, suggests that, if for the Levene test, the difference is significantly <0.05, then we have to consider the value 2 of p, and if the difference is not significant, we have to consider the value 1 of p. According to that, the difference between the two methods is not significant for fractions I (significance = 0.000) and III (significance = 0.002); p is 0.437 and 0.212 for fraction I and III respectively. However, the difference is significant for fraction II (significance = 0.146) and p is 0.003 for this fraction. This is probably due to the interference of proteins combined to GAGs that have not been completely eliminated, either by alkaline hydrolysis or by addition of TCA in fraction II, and which could be detected by CPC. This implies that enzymatic digestion alone applied during the extraction process, cannot generate stable fractions.

**Table 8:** Independent samples test of CS – DS chains contents by HPLC and CPC methods

		Levene test on the equality of variances		T-test for equality of mean						
		F	Sig.	t	ddl	Sig. (bilateral)	Average difference	Difference standard deviation	Confidence interval 95% of the difference	
									Lower	Upper
Fraction I	Assumption of equal variances	239.262	.000	1.964	4	.121	7.25000	3.69116	−2.99831	17.49831
	Assumption of unequal variances			1.216	1.009	.437	7.25000	5.96301	−66.97839	81.47839
Fraction II	Assumption of equal variances	3.815	.146	−8.709	3	.003	−9.21667	1.05826	−12.58451	−5.84882
	Assumption of unequal variances			−11.230	2.015	.008	−9.21667	.82074	−12.72316	−5.71018
Fraction III	Assumption of equal variances	49.020	.002	−3.863	4	.018	−5.40000	1.39777	−9.28082	−1.51918
	Assumption of unequal variances			−2.634	1.102	.212	−5.40000	2.04980	−26.36791	15.56791

HPLC method therefore remains the most accurate and safe method for the analysis of each GAG in a specific way; however, it cannot be applied to determine the mass of each molecule. It is often combined with mass spectrometry (MS) to identify and quantify eluted disaccharides [36–38].

## Discussion

A study of the same species “*Scyliorhinus canicula*”, showed that the CS is of type CS-A, CS-C, CS-D and CS-0S with the proportions 41, 32, 19.8 and 8.2%, respectively [9]. Dermatan sulfate was also the main glycosaminoglycan in the skin of *“Scyliorhinus canicula”* and represents 75% of the polysaccharides [39].

On GAGs preparation methods and after proteolysis, TCA treatment is commonly used to precipitate residual proteins and peptides [15, 16]. This procedure may not be necessary for the preparation of galactosaminoglycans for human consumption. Recent studies suggested that purity of CS isolated by anion exchange chromatography after chicken tissue digestion with papain was similar to samples treated with and without TCA [15]. This information may be useful for the preparation of CS for human consumption without the use of hazardous chemicals. In other tests, deproteinization with TCA is suggested as an important process [8]. The development of an UF-DF purification process has also been studied using ceramic membranes, and it demonstrates a purity superior to that of the ion exchange resin [40].

During the revelation and identification of GAGs by electrophoresis, the staining method combining toluidine blue / Stains All described by Volpi [41] has been used to stain complex sulphated GAGs (CS, DS, Hep, HS) as well as unsulfated ones (HA) which cannot bind to toluidine blue. The method has been used in many protocols. It can be used to stain low molecular weight fractions as well as oligosaccharides [42, 43]. In addition to its simplicity, this technique seems to produce a more stable and visible complex of GAGs and the two cationic dyes. In another study, Volpi has demonstrated that the common electrophoretic conditions used to separate compounds from a mixture of GAGs in agarose gel cannot separate HA and DS from each others without prior enzymatic depolymerisation [41]. According to our study, a clear segregation between molecules of GAGs was obtained on the electrophoretic profiles without prior enzymatic degradation.

NMR analysis of intact polymer was important because it excludes the appearance of rare sulfation units, such as those having O-^3^ sulfated GlcA, and / or oversulfated repeating units (OSCS) [9]. NMR has even been recommended by the Food and Drug Administration (FDA) as one of the analytical techniques for OSCS screening. After analysis, it was found that our extracted chondroitin sulfate molecule was devoid of toxic motifs.

For the titration of GAGs, in addition to the CPC reaction [25], the alcian blue method, the carbazole assay and the Dimethylmethylene Blue (DMMB) method, have also been used for GAGs quantification, but the validity of these methods largely depends on the purity of the GAG. The alcian blue assay is an easy way to quantify GAGs, but the latter detects only sulfated ones [44]. The carbazole assay consists of an estimation of the uronic acid content in CS. The absorbance of the carbazole-uronic acid colored complex is measured at 525 nm. The method was first reported by Dische [45] then modified [46, 47], and has been applied in several works. However, for this reaction, GAGs must be free of salts and other sugars such as glucose for accurate estimation. For the DMMB method, the reagent used is unstable. It can interfere with DNA and other negatively charged molecules and modify the GAGs measurements. In fact, these methods are simple and applicable in most research laboratories, but they are not specific for CS, and most of them determine the total GAGs amount [48]. In addition to that, titration with CPC has been used to characterize CS, however, this method cannot distinguish between CS and associated GAGs, and is subject to interference.

Numerous studies for the separation of GAGs after enzymatic degradation have been reported [49, 50]. These long and expensive approaches include: cellulose acetate membrane electrophoresis, polyacrylamide gel electrophoresis, anion exchange liquid chromatography (SAX-HPLC) and capillary electrophoresis. However, cellulose acetate membrane electrophoresis and polyacrylamide gel electrophoresis are laborious and tedious, and ion exchange HPLC requires a very expensive column.

HPLC is the most widely used technique in analytical laboratories and is frequently used in pharmacopoeia monographs. Several HPLC methods are available for the analysis of heparin, mainly anion exchange chromatography (SAX) or reverse phase chromatography. These methods have been used for isolation, purification and mapping of depolymerized heparin oligosaccharides [27, 51, 52].

On the other hand, GAGs cannot be detected reliably and accurately after enzymatic digestion. Indeed, several enzymes can hydrolyze at once several substrates, such as hyaluronidase [53] and chondroïtinase [54] that digest both CS and HA. In 1984, Kodama and al separated and quantified CS disaccharides by HPLC after enzymatic digestion of GAGs with chondroïtinase [55]. This technique is unaffected by the presence of salt or sulfation level and produces reproducible values ​​for all types of GAGs tested. These results suggest that HPLC test would generally be useful for routine analysis, in contrast to carbazole or alcian blue assay [56].

## Conclusion

The cartilage of « *Scyliorhinus canicula* » consists exclusively of CS and DS. The DS being predominant and represents 87.111%, while CS covers 12.889% of total GAGs of the cartilage. The FT-IR and electrophoresis identification tests showed that CS is of type C. The adopted extraction and purification process have the advantage of using a minimum of chemicals, however, deproteinization with TCA represents a necessary step, and additional steps should be considered for further purification, as the UF-DF method. The sequential ethanol extraction method yields two distinctly separated molecules with purity exceeding 90% for some fractions. The quantification methods showed that titrimetric CPC assay was not a reliable technique for determining CS content, although it is one of the tests adopted by the European Pharmacopoeia. In addition, the present study revealed that the HPLC method used for the direct quantification of CS, requires a prior identification of the GAGs ​​since it is not specific, despite its direct use in other studies. However, such method remains more accurate than titrimetric assay. The CS molecule extracted with TCA is conform to the tests required by the European Pharmacopoeia. Chondroitin sulfate, as well as Dermatan sulfate, were extracted from « *Scyliorhinus canicula* » co-product with the intention of using such bio-resource for polysaccharides recovery. In other studies, analyses have been concentrated to determine the chemical composition of CS, with particular attention to its sulfation model such rare patterns as those found in sea cucumber CS [57] or squid [58], which were excluded from our molecule according to the NMR analysis. According to the reported results, and taking into account that chemical composition of CS of this species is quite similar to that reported for shark cartilage, as well as the compliance of the CS with the tests of the European Pharmacopoeia, it is reasonable to consider «*Scyliorhinus canicula*» coproducts as an alternative source of CS and DS for nutritional and pharmacological applications [9].

## Methods

### Reference Substances and Reagents Used

Standards of Chondroitin sulfate and Dermatan sulfate are of USP grade. Hyaluronic Acid and Heparin are from SIGMA ALDRICH. Bovine albumen of LOBACHEMIE. All the products used are of reagent quality (SIGMA ALDRICH AND LOBACHEMIE).

### Preparation of Raw Material

Cartilage was obtained from the fish heads recovered from the local market. The procedure used to obtain cartilage powder involves a process of grinding and drying the raw material. The final product should be in the form of fine powder and maintains low moisture content.

### Extraction and Purification Process

In this study, 20 g of cartilage powder are suspended in 200 ml of 0.1 M sodium acetate buffer, 5 mM EDTA, 5 mM cysteine-HCl, pH 6. The enzymatic treatment is carried out by adding 80 mg of papain (Sigma -Aldrich, St. Louis, MO). The preparation is thoroughly mixed and then incubated for 24 h at 65 °C (optimal temperature for papain activity). Digestion is stopped by denaturing the enzyme at 100 °C for 10 min. The removal of the protein residues was carried out by centrifugation (Hettich 380R centrifuge, max speed 1500 rpm, made in Germany) and NaCl (2%) is added to the supernatant. Indeed, a sufficient concentration of salt was necessary for complete precipitation of the polysaccharides.

GAGs extracts were filtered through a membrane (Wathman 4 kDa) to remove salts and low molecular weight materials. The filtrates were then further purified and dialyzed (dialysis tubing cellulose membrane avg. flat width 33 mm, SIGMA-ALDRICH) against deionized water for 24 h at 4 °C, to provide crude polysaccharide fraction after removal of contaminants which persisted after filtration. A second dialysis was performed for 24 h after replacement of the dialysis water. The purified GAG solutions were lyophilized in a CHRIST type lyophilizer (Alpha 2–4 LDplus) until a completely dried material is obtained.

### Extraction of Total GAGs

In this study, three protocols are performed. In the first two tests, GAGs are extracted with and without TCA deproteinization to evaluate the possibility of omitting this step in the extraction protocol. TCA (7% of the total volume) was added to one of the solutions for deproteinization. After 24 h at 4 °C, centrifugation is carried out to remove the residual proteins. Two volumes of pure ethanol (99%) is added to the suspension of the two solutions, which will be kept at 4 °C for 24 h to allow the precipitation of polysaccharide components (GAGs). The sediment was collected by centrifugation (9000 rpm, 30 min, 4 °C). Fractions I (with TCA added) and II (without TCA added) of total GAGs were obtained.

In the third trial, total GAGs are extracted by enzymatic digestion followed by alkaline hydrolysis with NaoH 2 M (24 h at 40 °C, pH 9) to evaluate their ​​content after simple enzymatic digestion or combined hydrolysis. TCA is not used for this test. Fraction III of total GAGs was recovered. In all three processes, it was preferred not exceeding about two volumes of ethanol to avoid simultaneous precipitation of undesirable digests.

### Sequencial Precipitation of GAGs Fractions

In this test, total GAGs fraction was identical to fraction I. GAGs were fractionated by sequential precipitation with ethanol 99%. The volume of ethanol added is gradually increasing (from 0.2 to 2.0 volumes). For each 0.2 volume of ethanol, the mixture was kept at 4 °C for 24 h, then the precipitate was collected by centrifugation. Another 0.2 volume of ethanol was added to the supernatant, and the procedure was repeated to reach 2.0 volumes of ethanol. In total, 10 sediments were recovered after extraction (from A to J). The precipitates were suspended in ultrapure water to initiate the purification steps by filtration and dialysis.

### Physico-Chemical Characteristics of GAGs

Identification of GAGs and especially the extracted CS must meet several criteria. It involves a characterization of the chondroitin sulphuric acid anion by IR spectrum and CPC reaction; the sodium revelation by the sodium reaction; a verification of a sufficient salinization by measurement of pH; a verification of the limit levels in impurities by the chloride test and the determination of the contaminating proteins by the Lowry test. The European Pharmacopoeia described all these physico-chemical tests.

### pH Measurment

The pH of different GAGs solutions extracted must range between 5.5 and 7.5 [28]; the measurement was carried out using a meter METTLER TOLEDO Multiparameter Seven Excellence (made in Switzerland).

### Sodium Reaction

The identification of the sodium in the GAGs ​​extract solution was carried out by the sodium reaction described by the pharmacopoeia using methoxyphenylacetic reagent, ammonia and ammonium carbonate solution to precipitate residual sodium [28].

### Chloride Test

The chlorides of extracted GAGs are revealed by nitric acid and silver nitrate. In the dark, if the solution exhibits opalescence, it should not be more pronounced than that of the standard GAGs solution [28].

### Loss on Drying

The loss on drying (LOD) should not exceed 12.0% [28]. It is determined in an oven at 105 °C for 4 h per 1.000 g of test substance according to the following formula:

$$ LOD=\frac{\left( Ti+ Pe\right)- Tf}{Pe}\ X100 $$

Ti: initial tare / Tf: final tare / Pe: test portion.

An oven F10 A Vaccucell (France) and a balance Mettler Toledo type Newclassic MF (made in Switzerland MS Model 204 S / 01 max 220 g-min 0.1 mg) are used.

### Intrinsic Viscosity

The viscosity is determined at 25.00 ± 0.03 °C [28], using a suitable suspended level viscometer (specifications: viscometer constant = about 0.005 mm ^2^ / s ^2^). The relative viscosity ηr is determined from the ratio of the flow times « ti » obtained for the test solution to the flow time « t0 » obtained for the solvent, applying a kinetic energy correction related to the capillary (B = 30,800 s^3^) using the following expression:

$$ \upeta \mathrm{r}=\frac{\mathrm{t}\mathrm{i}-\frac{B}{ti^2}}{\mathrm{t}0-\frac{B}{t{0}^2}} $$

The specific viscosity ηs is deduced from the relative viscosity ηr using the following expression:

$$ \upeta \mathrm{s}=\upeta \mathrm{r}-1 $$

The concentration c (expressed in kg / m^3^) of chondroitin sodium sulphate in the test solution is determined using the following expression:

$$ \left[C\right]=m{0}_p\times \frac{x}{100}\times \frac{100-h}{100}\times 10 $$

x = percentage of CS content determined in the CPC assay.

h = loss on drying, in percent.

Intrinsic viscosity [η] is calculated from the regression line established by the least squares method using the following equation:

$$ \left[\eta \right]=\frac{\upeta \mathrm{s}}{c}=c\times {K}_H+\left[\upeta \right] $$

Ci = concentration of the test substance expressed in kg / m^3^.

K_H_ = Huggins constant.

### Protein Assay

The contaminating protein content should not exceed 3%. Quantification of contaminating proteins of extracted GAGs was performed according to the modified Lowry method [59]. This colorimetric analysis is validated, and did not differ from that of the monograph of the Ph. Eur. Briefly, three solutions were used for the determination of proteins: a solution containing potassium sodium tartrate and sodium carbonate (A), a solution containing potassium sodium tartrate and copper sulfate (B) and a freshly prepared Folin-Ciocalteu solution (C). The absorbance of the colored complex is measured at 650 nm.

### Qualitative Analysis of Glycosaminoglycan Extracts

#### Determination of Impurities by Agarose Gel Electrophoresis

The potential of electrophoresis to analyse GAGs and their derived oligosaccharides is excellent. It makes it possible to separate the different polysaccharides according to their density and their negative charge.

Agarose gel electrophoresis is performed according to the method described by the European Pharmacopoeia with some modifications. A NANOPAC-300 Cleaver scientific electrophoretic instrument is used. An agarose gel of about 4 to 5 mm, at a concentration of 0.5% in 0.001 M barium acetate buffer (buffered to pH 5 with acetic acid) was prepared. Three concentrations of CSC standard solution were applied (30, 0.6 and 0.3 mg/ml). 10 μl of GAGs samples are applied to the gel using a micropipette. After migration for 150 min at 300 V, the plate was soaked in ethanol-ultrapure water solution (30/70, v / v) and the GAGs ​​samples were fixed in the gel. The latter was dried and then stained with toluidine blue at 0.2% in acetic acid-ethanol-water (0.1/ 5/ 5, v/v) for 30 min, then decoloured with water to reveal sulfated GAGs. Additional Stains-All staining (25 mg in 500 ml ethanol-water 50/50 overnight in the dark) is performed to reveal the presence of HA.

#### Identification of GAGs by Fourier Transform Infrared (FT-IR) Spectroscopy

Fourier transform infrared (FT-IR) analysis is performed in transmission mode and was recorded using spectral scans in the range of 4000 to 800 cm-1. IR profiles were acquired using the PerkinElmer Spectrum Spotlight 100 ATR Imaging System (USA). Each standard GAG molecule (HA, HS, HEP, DS, C4S, C6S) as well as the extraction samples were independently analyzed as a powder using infrared microspectroscopy.

#### Identification of GAGs by Nuclear Magnetic Resonance (NMR) Spectroscopy

The analysis of the proton (1H nucleus) of extracted GAGs was analysed by NMR. Ten milligrams of each sample were introduced into a specific tube of 5 mm thick. The compound was dissolved in 500 μl of D_2_O (Deuterium oxide) inside the NMR tube. Standards were prepared in the same way for the analysis (10 mg of HA, CS and DS USP quality in 500 μl of D_2_O).

The proton NMR spectra of various samples were acquired through a BRUKER spectrometer equipped with a 500 MHz Ultrashield PLUS magnet, an AVANCE III console and using a wideband direct observation (BBFO) probe. The proton was acquired during a scan number NS = 16 scan, and at a temperature of 37 °C. The data was processed with the TOPSPIN 2.1 software and the spectra recorded. The results obtained for the samples were compared with those of the standards.

### Quantitative Analysis of Glycosaminoglycan Samples

#### Photometric Determination with Monohydrated Cetylpyridinium Chloride (CPC)

This quantification method is described by the European Pharmacopoeia [28] and is applied to chondroitin sulfate. The titration of GAGs ​​solution containing CS with cetylpyridinium chloride as a titrant was carried out with a METTLER TOLEDO T50 TA-02 ID Excellence Titrator Version 3.1.1 autotitrator equipped with a phototrode. The reading was performed at an appropriate wavelength in the visible region, and the percent content of CS is calculated according to the formula:

$$ \%\mathrm{CS}=\frac{V1\times m0}{V0\times m1}\times \frac{100}{100-h}\times Z $$

V0: volume of appropriate titrant reagent used for the appropriate control solution, in milliliters.

V1: volume of appropriate titrant reagent used for the appropriate test solution, in milliliters.

h: loss on desiccation of the test substance, in percent.

Z: % H_2_O (C_14_H_19_NNa_2_O_14_S) x Sodium Chondroitin Sodium SCR.

#### Quantification and Identification by HPLC Method

Quantitative High-Performance Liquid Chromatography is performed on HPLC (Waters) with UV-visible detector, using an XTerra column RP18, 5 μm, 4.6*250 mm. Chemicals used were from Sigma-ALDRICH, Carlo Erba and LOBA Chemie. Ultrapure HPLC grade water was prepared using a Millipore purification system. All solutions should be freshly prepared.

The LC buffer and the mobile phase were prepared as reported elsewhere [22]. The buffer is composed of triethylamine and orthophosphoric-acid in ultrapure water (10/8 to 100 ml). The mobile phase was composed of buffer and acetonitrile (5/40 to 1000 ml); 0.5 g octanesulfonic acid are added to this solution; pH 4 was regulated with buffer components including triethylamine and 85% orthophosphoric-acid. The solution is filtered through a 0.45 μm Millipore filter before use. Washing phase should be sonicated.

Operating conditions were applied as described, with some modifications. The method use an UV detection at 200 nm. Standards and sample solutions are prepared at a concentration of 75%. Samples are dissolved in mobile phase and sonicated until completely dissolved. This step helps prevent pH fluctuations, the peak areas of the injections are therefore stable and flow path chromatograms are better than when working in conditions of unadjusted pH. Solutions are clarified by filtration through 0.45 μm filter before injection. The system is equilibrated for 30 min and standard solutions are injected before sample analysis. The method is validated and is used in routine tests in our laboratory. The sodium sulfate content of chondroitin is given by the following formula:

$$ \% CS=\frac{Atest}{Acont}\times \frac{Wcont}{20}\times \frac{100}{Wtest}\times 100 $$

Atest: Peak area of CS in the chromatogram of the sample solution.

Acont: Peak area of CS in the chromatogram of the standard solution.

Wcont: weight of standard of CS in mg.

Wtest: weight of sample of CS in mg.

Standard: 100 ± 5% or 95.0 to 105.0%.

## Data Availability

Datasets and materials are available by the corresponding author.

## Abbreviations

CS: Chondroïtin Sulfate
DMMB: Dimethylmethylene Blue
DS: Dermatan Sulfate
FDA: Food and Drug Administration
FT-IR: Fourier Transform Infrared Spectroscopy
GAGs: Glycosaminoglycans
HA: Hyaluronic Acid
Hep: Heparin
HPLC: Hight Performance Liquid Chromatography
HS: Heparan Sulfate
KS: Keratan Sulfate
LOD: Loss On Drying
NMR: Nuclear Magnetic Resonance
OSCS: Over Sulfated Chondroïtin Sulfate
Ph. Eur.: European Pharmacopeia
USP: United States Pharmacopeia

## References

[1] Silva TH, Alves A, Ferreira BM, Oliveira JM, Reys LL, Ferreira RJF, and al (2012). Materials of marine origin : a review on polymers and ceramics of biomedical interest. Int Mater Rev.

[2] Liu Z, Zhang F, Li L, Li G, He W, Linhardt RJ (2014). Compositional analysis and structural elucidation of glycosaminoglycans in chicken eggs. Glycoconj J.

[3] Reginster J-Y, Dudler J, Blicharski T, Pavelka K (2017). Pharmaceutical-grade chondroitin sulfate is as effective as celecoxib and superior to placebo in symptomatic knee osteoarthritis : the ChONdroitin versus CElecoxib versus placebo trial (CONCEPT). Ann Rheum Dis.

[4] Albertini R, De Luca G, Passi A, Moratti R, Abuja PM (1999). Chondroitin-4-sulfate protects high-density lipoprotein against copper-dependent oxidation. Arch Biochem Biophys.

[5] Campo GM, Avenoso A, Campo S, Ferlazzo AM, Micali C, Zanghí L, and al (2004). Hyaluronic acid and chondroitin-4-sulphate treatment reduces damage in carbon tetrachloride-induced acute rat liver injury. Life Sci.

[6] Uebelhart D, Malaise M, Marcolongo R, DeVathaire F, Piperno M, Mailleux E, and al (2004). Intermittent treatment of knee osteoarthritis with oral chondroitin sulfate : a one-year, randomized, double-blind, multicenter study versus placebo. Osteoarthritis Cartilage.

[7] Volpi N (2009). Quality of different chondroitin sulfate preparations in relation to their therapeutic activity. J Pharm Pharmacol.

[8] Nakano T, Pietrasik Z, Ozimek L, Betti M (2012). Extraction, isolation and analysis of chondroitin sulfate from broiler chicken biomass. Process Biochem.

[9] Gargiulo V, Lanzetta R, Parrilli M, De Castro C (2009). Structural analysis of chondroitin sulfate from Scyliorhinus canicula : a useful source of this polysaccharide. Glycobiology..

[10] Kim S-B, Ji C-I, Woo J-W, Do J-R, Cho S-M, Lee Y-B, and al (2012). Simplified purification of chondroitin sulphate from scapular cartilage of shortfin mako shark (*Isurus oxyrinchus*) : Simple purification of chondroitin sulphate. Int J Food Sci Technol.

[11] Xie J, Ye H-Y, Luo X-F (2014). An efficient preparation of chondroitin sulfate and collagen peptides from shark cartilage. Int Food Res J.

[12] Murado MA, Fraguas J, Montemayor MI, Vázquez JA, González P (2010). Preparation of highly purified chondroitin sulphate from skate (Raja clavata) cartilage by-products. Process optimization including a new procedure of alkaline hydroalcoholic hydrolysis. Biochem Eng J.

[13] Nakano T, Ikawa N, Ozimek L (2000). An economical method to extract chondroitin sulphate-peptide from bovine nasal cartilage. Can Agric Eng.

[14] Nakano T, Nakano K, Sim JS (1998). Extraction of glycosaminoglycan peptide from bovine nasal cartilage with 0.1 M sodium acetate. J Agric Food Chem.

[15] Nakano T, Betti M, Pietrasik Z (2010). Extraction, isolation and analysis of chondroitin sulfate glycosaminoglycans. Recent Pat Food Nutr Agric.

[16] NAKANO T, SIM JS (1995). A study of the chemical composition of the proximal Tibial articular cartilage and growth plate of broiler chickens. Poult Sci.

[17] Shetty AK, Kobayashi T, Mizumoto S, Narumi M, Kudo Y, Yamada S (2009). And al. Isolation and characterization of a novel chondroitin sulfate from squid liver integument rich in N-acetylgalactosamine (4,6-disulfate) and glucuronate (3-sulfate) residues. Carbohydr Res.

[18] Volpi N (1996). Purification of heparin, dermatan sulfate and chondroitin sulfate from mixtures by sequential precipitation with various organic solvents. J Chromatogr B.

[19] Paulsen BS, Olafsdóttir ES, Ingólfsdóttir K (2002). Chromatography and electrophoresis in separation and characterization of polysaccharides from lichens. J Chromatogr A.

[20] Blanco M, Fraguas J, Sotelo C, Pérez-Martín R, Vázquez J (2015). Production of chondroitin Sulphate from head, skeleton and fins of Scyliorhinus canicula by-products by combination of enzymatic, chemical precipitation and ultrafiltration methodologies. Marine Drugs.

[21] Mansour MB, Dhahri M, Hassine M, Ajzenberg N, Venisse L, Ollivier V, and al (2010). Highly sulfated dermatan sulfate from the skin of the ray *Raja montagui* : Anticoagulant activity and mechanism of action. Comp Biochem Physiol B Biochem Mol Biol.

[22] Tyler T, Khandelwal B, Norden D, Rolle F-R (2002). Determination of chondroitin sulfate in raw materials by liquid chromatography. J AOAC Int.

[23] Volpi N, Maccari F, Titze J (2005). Simultaneous detection of submicrogram quantities of hyaluronic acid and dermatan sulfate on agarose-gel by sequential staining with toluidine blue and stains-all. J Chromatogr B.

[24] Volpi N (2007). Analytical aspects of pharmaceutical grade chondroitin sulfates. J Pharm Sci.

[25] Liang Z, Bonneville C, Senez T, Henderson T (2002). Development and validation of a photometric titration method for the quantitation of sodium chondroitin sulfate (bovine) in Cosequin DS chewable tablet. J Pharm Biomed Anal.

[26] N Periyasamy, S Murugan, P Bharadhirajan (2013). Isolation and characterization of anticoagulant compound from marine mollusc Donax faba (Gmelin, 1791) from Thazhanguda, Southeast Coast of India. African Journal of Biotechnology.

[27] Beni S, Limtiaco JFK, Larive CK (2011). Analysis and characterization of heparin impurities. Anal Bioanal Chem.

[28] Council of Europe. European Pharmacopoeia Commission. European pharmacopoeia. 9th ed. Strasbourg; 2007. 2017. (Supplement 9.2 ; vol. 1

[29] European pharmacopeia. Chondroitin sulfate sodium monograph. In European Pharmacopeia, 7th ed. Strasbourg: European Directorate for the Quality of Medicines; 2007 p. 1681–1683.

[30] Didraga M, Barroso B, Bischoff R (2006). Recent developments in proteoglycan purification and analysis. Curr Pharm Anal.

[31] Ben Mansour M, Dhahri M, Bertholon I, Ollivier V, Bataille I, Ajzenberg N (2009). And al. Characterization of a novel dermatan sulfate with high antithrombin activity from ray skin (Raja radula). Thromb Res.

[32] Khan H, Ashraf M, Saeed Hashmi A, Ahmad M-D, Anjum A (2013). Extraction and biochemical characterization of Sulphated Glycosaminoglycans from chicken keel cartilage. Pak Vet J.

[33] Brézillon S, Untereiner V, Lovergne L, Tadeo I, Noguera R, Maquart F-X (2014). And al. Glycosaminoglycan profiling in different cell types using infrared spectroscopy and imaging. Anal Bioanal Chem.

[34] Jagadeeswara Reddy K, Karunakaran KT (2013). Purification and characterization of hyaluronic acid produced by Streptococcus zooepidemicus strain 3523-7. J Biosci Biotech.

[35] Pomin VH (2014). NMR chemical shifts in structural biology of glycosaminoglycans. Anal Chem.

[36] Yoshida K, Miyauchi S, Kikuchi H, Tawada A, Tokuyasu K (1989). Analysis of unsaturated disaccharides from glycosaminoglycuronan by high-performance liquid chromatography. Anal Biochem.

[37] Kinoshita A, Sugahara K (1999). Microanalysis of glycosaminoglycan-derived oligosaccharides labeled with a Fluorophore 2-Aminobenzamide by high-performance liquid chromatography : application to disaccharide composition analysis and exosequencing of oligosaccharides. Anal Biochem.

[38] Kubaski F, Osago H, Mason RW, Yamaguchi S, Kobayashi H, Tsuchiya M, and al (2017). Glycosaminoglycans detection methods : Applications of mass spectrometry. Mol Genet Metab.

[39] Dhahri M, Mansour MB, Bertholon I, Ollivier V, Boughattas NA, Hassine M (2010). Anticoagulant activity of a dermatan sulfate from the skin of the shark Scyliorhinus canicula. Blood Coagul Fibrinolysis.

[40] Lignot B, Lahogue V, Bourseau P (2003). Enzymatic extraction of chondroitin sulfate from skate cartilage and concentration-desalting by ultrafiltration. J Biotechnol.

[41] Volpi N, Maccari F (2002). Detection of submicrogram quantities of glycosaminoglycans on agarose gels by sequential staining with toluidine blue and stains-all. ELECTROPHORESIS.

[42] Dietrich CP, Dietrich SMC (1976). Electrophoretic behaviour of acidic mucopolysaccharides in diamine buffers. Anal Biochem.

[43] Volpi N, Maccari F (2005). Microdetermination of chondroitin sulfate in normal human plasma by fluorophore-assisted carbohydrate electrophoresis (FACE). Clin Chim Acta.

[44] Frazier SB, Roodhouse KA, Hourcade DE, Zhang L (2008). The quantification of Glycosaminoglycans : a comparison of HPLC, Carbazole, and Alcian blue methods. Open Glycosci.

[45] Dische Z (1947). A new specific color reaction of hexuronic acids. J Biol Chem.

[46] Bitter T, Muir HM (1962). A modified uronic acid carbazole reaction. Anal Biochem.

[47] Kosakai M, Yosizawa Z (1979). A partial modification of the carbazole method of bitter and muir for quantitation of hexuronic acids. Anal Biochem.

[48] Barbosa I (2003). Improved and simple micro assay for sulfated glycosaminoglycans quantification in biological extracts and its use in skin and muscle tissue studies. Glycobiology.

[49] Volpi N, Maccari F (2003). Purification and characterization of hyaluronic acid from the mollusc bivalve Mytilus galloprovincialis. Biochimie..

[50] Volpi N, Maccari F, Galeotti F, Zampini L, Santoro L, Padella L, and al (2013). Plasmatic dermatan sulfate and chondroitin sulfate determination in mucopolysaccharidoses. J Pharm Biomed Anal.

[51] Chuang W-L, McAllister H, Rabenstein DL (2001). Chromatographic methods for product-profile analysis and isolation of oligosaccharides produced by heparinase-catalyzed depolymerization of heparin. J Chromatogr A.

[52] Rice KG, Kim YS, Grant AC, Merchant ZM, Linhardt RJ (1985). High-performance liquid chromatographic separation of heparin-derived oligosaccharides. Anal Biochem.

[53] Sugahara K, Tanaka Y, Yamada S (1996). Preparation of a series of sulfated tetrasaccharides from shark cartilage chondroitin sulfate D using testicular hyaluronidase and structure determination by 500 MHz1H NMR spectroscopy. Glycoconj J.

[54] Huang W, Lunin VV, Li Y, Suzuki S, Sugiura N, Miyazono H, and al (2003). Crystal structure of Proteus vulgaris chondroitin sulfate ABC lyase I at 1.9A resolution. J Mol Biol.

[55] Kodama C, Ototani N, Isemura M, Yosizawa Z (1984). High-performance liquid chromatography of pyridylamino derivatives of unsaturated disaccharides produced from chondroitin sulfate isomers by chondroitinases. J Biochem.

[56] Campo GM, Campo S, Ferlazzo AM, Vinci R, Calatroni A (2001). Improved high-performance liquid chromatographic method to estimate aminosugars and its application to glycosaminoglycan determination in plasma and serum. J Chromatogr B Biomed Sci Appl.

[57] Vieira R-P, Mulloy B, Mourão P-A (1991). Structure of a fucose-branched chondroitin sulfate from sea cucumber. Evidence for the presence of 3-O-sulfo-beta-D-glucuronosyl residues. J Biol Chem.

[58] Habuchi O, Sugiura K, Kawai N (1977). Glucose branches in chondroitin sulfates from squid cartilage. J Biol Chem.

[59] Hartree EF (1972). Determination of protein : a modification of the Lowry method that gives a linear photometric response. Anal Biochem.

